# Fiscal space for Health in the Americas: is economic growth sufficient?*

**DOI:** 10.26633/RPSP.2018.321

**Published:** 2019-10-30

**Authors:** Camilo Cid Pedraza, Mauricio Matus-López, Ernesto Báscolo

**Affiliations:** 1 Department of Health Systems and Services Pan American Health Organization Washington DC United States of America Department of Health Systems and Services. Pan American Health Organization, Washington DC, United States of America.

**Keywords:** Healthcare financing, universal coverage, health resources, Latin America, Financiación de la atención de la salud, cobertura universal, recursos en salud, América Latina, Financiamento da assistência à saúde, cobertura universal, recursos em saúde, América Latina

## Abstract

**Objective.:**

In 2014, the member countries of the Pan American Health Organization signed the Strategy for Universal Access to Health and Universal Health Coverage. In it, they committed to increasing public expenditure on health until reaching the benchmark of 6% of gross domestic product (GDP). The objective of this paper is to determine, for each country in the Region, whether they can reach this target through economic growth alone and, if so, how long it would take.

**Methods.:**

Using World Bank and World Health Organization data, elasticity of public health expenditure (PHE) with respect to GDP was estimated for each country. Real economic growth and International Monetary Fund projections for 2016–2021 were used to project the expenditure series and determine the year each country would reach 6% of GDP.

**Results.:**

Six countries have already reached the 6% target. The Latin American and Caribbean countries that have achieved it are those that have single health systems, based on universal access and coverage. If current prioritization of PHE is maintained, three countries could reach the target in the next decade. Four more countries would reach it before mid-century, 10 in the second half of the century, and one would have to wait until the next century. Finally, 13 countries would never reach the proposed target.

**Conclusions.:**

This analysis demonstrates the limitations of economic growth as a source of fiscal space. Other sources will need to be tapped, such as increased tax collection, specific health taxes, and greater efficiency in public spending, which will require social and political dialogue in the countries regarding their commitment to universal health principles.

## RESUMEN

**Objetivo.:**

En 2014, los países miembros de la Organización Panamericana de la Salud firmaron la Estrategia para el acceso universal a salud y cobertura universal de salud. En ella, se comprometieron a aumentar el gasto público en salud hasta alcanzar la meta referencial de 6% del producto interno bruto (PIB). El objetivo de este trabajo es determinar, para cada uno de los países de la Región, si pueden alcanzar esta meta solo con crecimiento económico y, en el caso de ser posible, en qué plazos lo harían.

**Métodos.:**

Se utilizaron datos del Banco Mundial y de la Organización Mundial de Salud y se estimaron las elasticidades del gasto público en salud con respecto al PIB para cada país. Con base en el crecimiento económico real y el proyectado por el Fondo Monetario Internacional 2016–2021, se proyectó la serie de gasto y se determinó el año en el que alcanzarían 6% del producto.

**Resultados.:**

Seis países ya han alcanzado la meta de 6%. Los países de América Latina y el Caribe que la han logrado son aquellos que mantienen sistemas de salud únicos, basados en acceso y cobertura universales. Si se mantiene la priorización actual del gasto público en salud, tres países podrían alcanzar la meta en la próxima década. Otros cuatro países lo harían antes de medio siglo, diez en la segunda mitad y uno tendría que esperar hasta la próxima centuria. Por último, 13 países nunca alcanzarían la meta propuesta.

**Conclusiones.:**

Este análisis demuestra las limitaciones del crecimiento económico como fuente de espacio fiscal. Será necesario recurrir a otras fuentes como mayor recaudación tributaria, impuestos específicos en salud y mayor eficiencia en el gasto público, lo que demandará un diálogo social y político de los países en torno al compromiso con los principios de la salud universal.

## RESUMO

**Objetivo.:**

Em 2014, os Estados Membros da Organização Pan-Americana da Saúde firmaram a Estratégia para o acesso universal à saúde e cobertura universal de saúde com a qual se comprometeram a aumentar o gasto público em saúde até atingir a meta de referência de 6% do produto interno bruto (PIB). O objetivo deste estudo foi determinar se cada um dos países da Região conseguiria atingir esta meta apenas com o crescimento econômico e, neste caso, em que prazo.

**Métodos.:**

O estudo se baseou em dados obtidos do Banco Mundial e da Organização Mundial de Saúde (OMS). Foi estimada a elasticidade do gasto público em saúde com relação ao PIB para cada país. A partir do crescimento econômico real e do crescimento projetado pelo Fundo Monetário Internacional para o período 2016–2021, foi feita a projeção dos gastos e determinado o ano em que seriam alcançados 6% do PIB.

**Resultados.:**

Seis países já atingiram a meta de 6%. Os países da América Latina e Caribe que atingiram esta meta são os que têm um sistema de saúde único baseado no acesso e cobertura universais. Se for mantida a priorização atual do gasto público em saúde, três países conseguiriam alcançar a meta na próxima década. Outros quatro países atingiriam a meta antes de meados do século, 10 na segunda metade deste século e um somente a alcançaria no século seguinte. E, por fim, 13 países nunca atingiriam a meta proposta.

**Conclusões.:**

Esta análise demonstra as limitações do crescimento econômico como fonte de espaço fiscal. Será necessário recorrer a outras fontes, como maior arrecadação tributária, impostos próprios para a saúde e maior eficiência no gasto público, o que demanda dos países um diálogo social e político quanto ao compromisso com os princípios de saúde universal.

The objective of the Universal Health Strategy of the Pan American Health Organization (PAHO) is to build a path toward universal access to health and universal health coverage (1-4). To accomplish this, a number of simultaneous interventions are linked with the need for public financing to ensure equitable access to health (2). In 2014, PAHO’s member states agreed to increase public health expenditure (PHE) to 6% of gross domestic product (GDP) (4). This implies an average increase in PHE of almost two and a half percentage points, which would considerably reduce the incidence of financial catastrophe and impoverishment of households due to out-of-pocket spending on health (5).

The importance of public expenditure on health has long been addressed, from the original studies on investment in social capital (6, 7) to the development of the social determinants of health (8). The most recent works confirm the long-term relationship between improvements in health, economic growth, and PHE (9–12).

Within this general framework, the importance of the concept of fiscal space for health (FSH) is clear. The first works on fiscal space (13, 14) addressed the need for more resources for health in middle- and low-income countries. These studies and those that followed defined FSH as a country’s capacity to generate resources to complement the public budget, without damaging the government’s finances or imperiling economic stability (15, 16).

A number of specific sources of potential FSH were proposed. Empirical studies grouped these into five categories: a) economic growth, b) greater tax revenue, c) budget reallocation, d) efficiency of expenditure, and e) resources received from abroad (16, 17).

The first and most studied of these sources is economic growth. The idea is that if GDP grows, the State’s tax collection will also grow. And if the proportional distribution of the budget remains unchanged, resources for health will increase. Although later studies have shown its limitations (18–21), this remains one of the main sources of financing in low- and middle-low-income countries.

The importance of economic growth was confirmed, both directly and indirectly, in a systematic review of 44 countries by the World Health Organization (22), in which this source was addressed in three ways. The majority of the studies assessed macroeconomic and fiscal conditions (18–21, 23). A second way of looking at the relationship between FSH and growth relied on estimates of public revenue made by the governments themselves, although these estimates were available for periods of only two or three years. A third group of studies attempted to quantify the increase in PHE with respect to GDP on a horizon of five years or less (24–26). However, none of these studies evaluated to what point economic growth can help countries to reach a specific target for PHE. This is the new contribution made by this paper.

In Latin America, country-specific studies confirm that economic growth is the most technically and politically feasible source of FSH (26–28). However, it is insufficient to achieve the PAHO target, at least on a five-year horizon, making it necessary to obtain funds from sources other than fiscal space.

The objective of this work is to determine, for each country of the Region of the Americas, whether it is possible to achieve the target of *PHE = 6% of GDP* solely through economic growth and, if so, in what year this would happen.

## MATERIALS AND METHODS

A quantitative, exploratory, and longitudinal study was conducted, with the sources and analyses presented in this paper.

### Sources

Three open sources were used. The first was the WHO health financing database (29). This provided the annual series of PHE as a percentage of GDP for the period 1995–2014. PHE corresponds to current expenditures and capital expenditures by central and local governments, as well as budgets, foreign loans and aid (including donations from international agencies and nongovernmental organizations that from part of a country’s public budget), and compulsory social insurance health funds. The second source was the World Bank database of development indicators (30). This showed the evolution of annual real GDP for each country for the period 1995–2015. Finally, International Monetary Fund projections for annual GDP growth (31) for the period 2016–2021 were used as the third source.

### Estimate

The statistical analysis was carried out in five steps. The first was to obtain the PHE series and exclude those countries that had exceeded the 6% GDP target in the most recent year with available information (2014).

In the second step, PHE elasticities with respect to GDP were calculated for the period 1995–2014. Atypical values were eliminated for each country, with a value of ± 1.5 of the interquartile range.

In the third step, 13 countries whose elasticities were less than 1 were excluded; i.e., PHE would not increase with respect to GDP in any scenario.

In the fourth step, PHE in the remaining countries was predicted according to the growth expected for 2016–2021, and historical average growth from then on.

Thus, the estimated PHE as a percentage of GDP was expressed in equation (a), as:


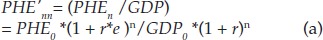


Where


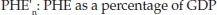


n: years after year 0

r: rate of annual growth in GDP, based on IMF projections for 2016–2021, and the historical rate for the following years

e: elasticity in PHE with respect to GDP.

Finally, in the fifth step, the number of years needed to reach the target of PHE = 6% of GDP was calculated as follows:


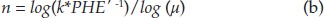


Where


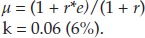


## RESULTS

Only five countries in the Region have PHE higher than 6% of GDP: Canada, Costa Rica, Cuba, the United States of America, and Uruguay. The other 30 countries fall short of the target. Of these, Colombia, Nicaragua, and Panama are within less than one point of reaching it. Seven other countries are more than two percentage points short, and nine are less than halfway to the target (Table 1).

The projections based on historical trends are not encouraging. Although the Region has grown at an average of 2.9% a year over the last 25 years, PHE has barely increased with respect to GDP. The result of this is that less than half of the countries (13) show elasticity less than 1.

In the next decade, only Bolivia, Colombia, and Ecuador could reach the target of PHE = 6% of GDP, while Chile, the Dominican Republic, El Salvador, and Nicaragua, would do so in the following decade. Peru would have to wait until the 22^nd^ century and the rest would not reach the target through economic growth alone.

**TABLE 1:** Main parameters of estimated public health expenditure, and year in which each country would reach the target of 6% of gross domestic product

Country	PHE 2014	Elasticity	Gap	Year
Countries that have already reached the target				
Canada	7.4	NA	NA	1995
United States of America	8.3	NA	NA	2001
Costa Rica	6.8	NA	NA	2002
Uruguay	6.1	NA	NA	2004
Cuba	10.6	NA	NA	2005
**Countries that could reach the target before 2030**				
Colombia	5.4	2.36	0.59	2024
Ecuador	4.5	3.30	1.49	2026
Plurinational State of Bolivia	4.6	2.20	1.43	2027
Dominican Republic	2.9	2.61	3.07	2029
Countries that could reach the target between 2030 and 2100				
Nicaragua	5.1	1.64	0.90	2031
El Salvador	4.5	2.13	1.53	2033
Chile	3.9	1.97	2.15	2035
Dominica	3.8	1.92	2.23	2052
Mexico	3.3	1.68	2.74	2056
Panama	5.9	1.10	0.12	2060
Brazil	3.8	1.49	2.17	2064
Paraguay	4.5	1.28	1.50	2068
Trinidad and Tobago	3.2	1.32	2.83	2068
Guatemala	2.3	1.62	3.67	2069
Haiti	1.6	2.95	4.44	2072
Honduras	4.4	1.17	1.58	2091
Peru	3.3	1.00	2.68	After 2100
**Countries than would never reach the target through economic growth alone**				
Antigua and Barbuda	3.8	<1	2.22	Never
Argentina	2.7	<1	3.35	Never
Bahamas	3.6	<1	2.40	Never
Barbados	4.7	<1	1.26	Never
Belize	3.9	<1	2.12	Never
Grenada	2.8	<1	3.17	Never
Guyana	3.1	<1	2.88	Never
Jamaica	2.8	<1	3.19	Never
Bolivarian Republic of Venezuela	1.5	<1	4.46	Never
Saint Vincent and the Grenadines`	4.4	<1	1.61	Never
Saint Lucia	3.6	<1	2.40	Never
St. Kitts and Nevis	2.1	<1	3.86	Never
Suriname	2.9	<1	3.06	Never

NA: not applicable

*Source:* the authors.

## DISCUSSION

Growth in the Region has been relatively high. Between 1995 and 2015, it averaged 3.2% a year (30) and projections for 2016–2021 put it in at an annual average of 2.5% (31). However, 13 countries will never reach the target of PHE = 6% of GDP through economic growth alone, and 10 more countries will have to wait until after 2050.

**FIGURA 1:**
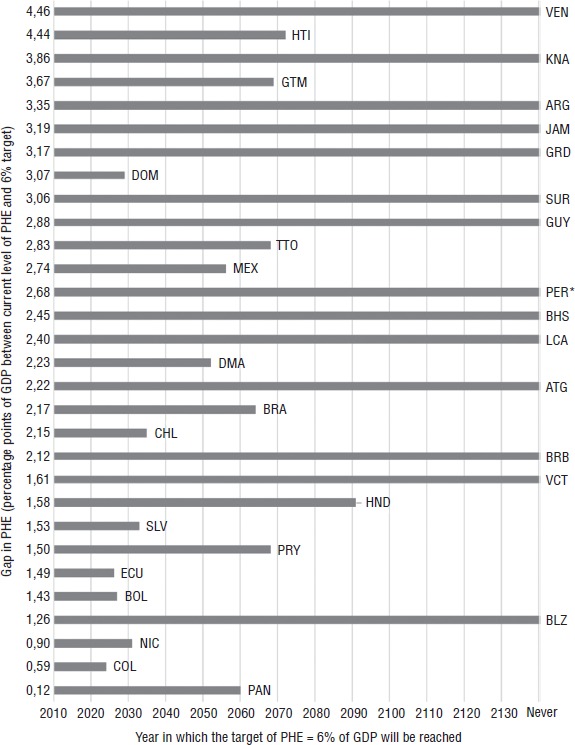
Countries by gap between current and target PHE, and year in which the economic growth target would be reached

The gap between current PHE and the 6% target is important, but not the determining factor. Countries with large gaps, such as Dominica or Guatemala, could come closer to the target if they maintain the revenue elasticity registered to date. A special case is Haiti, which has followed the same pattern, but has seen exceptional growth in PHE in recent years. In contrast, countries that are close to the target, such as Belize, will not increase their expenditure to the agreed level through economic growth alone (Figure 1).

These results coincide with analyses of fiscal space for health in international case studies (17–18, 22–24). Economic growth is essential in order to increase PHE, but PHE must be elastic with respect to GDP and economic growth must be maintained.

In the cases where PHE is inelastic, the capacity to create FSH is limited.

*After year 2100.

*Source:* the authors.

### Analysis of the results by groups of countries

The countries that have reached the target are characterized by one of two fundamental situations (PAHO/WHO, 2017) (32): developed countries such as Canada and the United States are in the group of countries that long ago surpassed the target; the others are countries that either maintained unified, integrated health systems with universal coverage and universal access or else transformed their health systems in this direction. This is the case of Costa Rica and Cuba, both of which have had universal systems since the middle of the 20^th^ century. Cuba, as is well known, began developing its national health system in the 1960s as one of its key social policies. Costa Rica, for its part, has operated the Costa Rican Social Security Fund since 1941 as a pooled fund; in the 1960s insurance was universalized to cover the entire population and in the 1990s it was consolidated as the single fund for the country’s universal health system. Finally, Uruguay is the most recent country to reach the indicator. At the same time, it has made great progress in universalizing its health system through a reform that began in 2008, characterized by a significant mitigation of segmentation. It should be noted that these countries are among those with the best health outcomes in the Region.

The second group is made up of countries that would achieve the target before 2030. Bolivia and Ecuador have been making major public investments in recent years as the result of policies that deliberately aim for universal and equitable access through the development of their model of care, while strengthening governance (through implementation of the Unified Intercultural and Community Health System and the Comprehensive Health Care Model, respectively). Colombia and the Dominican Republic are cases that need to be analyzed in greater detail, since the insurance- and coverage-related reforms implemented in both countries (in the 2000s in the Dominican Republic, and in the 1990s in Colombia, through Law 100) do not clearly indicate a strategic line of development for public investment.

In the group of 13 countries that would reach the target at some point, a subgroup of three countries would do so before 2035 (and could, therefore, be included in the previous group). El Salvador and Nicaragua have enacted policies in recent years that explicitly aim to increase access and strengthen governance. In El Salvador, this includes the elimination of copayments. Chile’s per capita health expenditure quadruples both those countries and it has made reforms; however, it is a more nuanced case with respect to PHE, with periods in which the indicator declined or stabilized, and other periods in which it increased, but not surpassing 4% of GDP.

The other subgroup remains much further from achieving the target (not until the year 2100) but it is not characterized by any common pattern. Some countries have made attempts to strengthen their health systems and have achieved progress through certain policies. These include Peru, which has improved coverage and access; Brazil, whose national unified health system (known as SUS) has high levels of access; and Mexico, which has attempted to improve coverage through broad-based insurance; as well as countries that have had problems consolidating their systems, such as Guatemala.

Finally, the countries whose elasticity in health expenditure with respect to GDP growth is less than 1 and which will never reach the target through economic growth alone are mainly in the non-Latin Caribbean. On average, they have low, stable PHE with respect to GDP and high out-of-pocket expenditure. Some of them have gone through or are still experiencing macroeconomic problems such as high indebtedness and have been subject to adjustment plans in recent years.

Experiences in the Region, as well as documented experiences elsewhere (33), show that in order to achieve the successes required to reach the target, it is necessary to have both political will at the highest level and social consensus on the need to improve the population’s health through decisive measures and policies.

For countries that have reached the target, the challenge is to maintain their achievements and make them sustainable. For those near the target, the challenge is to maintain their levels of PHE growth, even when facing problems in the business cycle such as those recently experienced, or changes in the political context often linked to shifting perspectives in government.

Unequal progress has been made with the different components of financing in the process of strengthening or transforming health systems. Experience shows that in order to lay the groundwork for greater progress, it is also necessary to make use of common, basic, financial instruments such as increased tax collection, leveraging the potential of pooled funds, understanding costs, effective budgetary planning, and more incentives to improve efficiency in resource allocation, among other measures (33).

## CONCLUSIONS

The country-specific studies show that economic growth has technical potential and is a politically feasible way to increase public health expenditure in the Region. However, there are limitations, given the trends in growth and the relationship between the variables of growth and PHE. In this study, the results are clear: few countries can reach the target of PHE = 6% of GDP based solely on resources generated by economic growth. For almost half of the countries, growth alone is insufficient to increase PHE; and for a third of them, the contribution of growth is so limited that they would have to wait until the second half of this century to achieve the target.

As other studies have concluded, this leads to a search for additional sources of fiscal space for health, such as changes in the tax rate (34–35). It will also be necessary to analyze other complementary sources. In Latin America, this would seem to suggest focusing on the currently low level of tax collection and seeking greater efficiency in public expenditure, among other measures (26–28). These changes will require broad-based social and political dialogue regarding the commitment to advancing toward universal health.

The limitations of this work have to do mainly with issues that go beyond the scope of this article. The first is that, beyond public health expenditure, each country faces other specific, pressing problems as it seeks to achieve universal health, such as the organization of health systems, governance, and medical management, among others. More resources for the sector will not, by themselves, eliminate inefficiencies or inequities in the national systems. The second limitation is that the target of PHE = 6% of GDP was set for the countries of the Americas in a formal agreement among PAHO’s member states in 2014. Although it has technical backing, achieving this target does not ensure sufficient resources for the achievement of universal health in each country.

Nevertheless, the analysis of each group shows that the countries that have health systems with universal coverage and access are those that have reached the target, and that those close to the target have implemented policies aimed at reforming or strengthening the health sector for several years.

Finally, the sources used have two types of limitations. First, updating the PHE series is a slow process that can take several years and can vary due to methodological differences, such as whether or not mandatory contributions are included. In a few cases, there are surprising changes in the data over time, for example in Argentina, and it is likely that this type of difficulty is involved. Furthermore, five-year projections of economic growth can vary as updates become available.

### Conflict of interests.

None declared by the authors.

### Declaration.

The opinions expressed in this manuscript are the responsibility of the authors and do not necessarily reflect the criteria or policies of ***PSP/PAJPH*** and/or PAHO.
